# The Impact of COVID-19 Outbreak on Camping Tourism in Spain: A Spatial Approach to Tourist Destinations

**DOI:** 10.1007/s41742-022-00474-x

**Published:** 2022-09-23

**Authors:** Francisca Jesús Sánchez-Sánchez, Ana María Sánchez-Sánchez

**Affiliations:** Departamento de Economía, Métodos Cuantitativos E Historia Económica, Universidad Pablo de Olavide, ES-41013 Sevilla, España

**Keywords:** Camping tourism, Nature destinations, Coastal destinations, COVID-19

## Abstract

The COVID-19 health crisis has wreaked devastation on the world economy, especially on the tourism sector. The camping sector has been little studied despite its high economic impact and participation rate. Moreover, the observable effects of phenomena such as the COVID-19 pandemic have received little research attention. Consequently, the objective of this paper is therefore to analyse the effects of the pandemic on camping tourism by characterising the factors that determine it. The study is carried out by providing a geographical perspective of the sector by tourist areas, whereby two types of tourist destinations are considered: campsites located in coastal areas, and campsites located in natural areas. This is the main contribution of the work, as the proposed geographical analysis studies smaller territorial units than those usually used in tourism research. For the study, Multivariate Analysis techniques are applied, specifically Factor Analysis and Cluster Analysis. The results show that there is a balance between supply and demand in the sector, with a significant economic impact, especially on employment and the performance of the sector. The impact of the COVID-19 pandemic has led to nature tourism gaining greater popularity, and shows an evolution in travellers' preferences for tourist destinations in favour of campsites located in natural areas over those located in coastal destinations. The geographical location of the tourist destination, therefore, plays a key role in the characterisation of Spanish campsites. This has practical implications for both camping companies and institutions, as the fact that some areas are more attractive than others is a decisive factor in deciding on the location of new campsites.

## Introduction

On 12 March 2020, the World Health Organisation (WHO) declared Coronavirus Disease 2019 (COVID-19) a pandemic. The pandemic has affected every aspect of our lives (Nicola et al. 2020); for example, it has influenced the way we live, communicate, work, and travel (Committee for the Coordination of Statistical Activities 2020). Exceptional measures imposed by governments have resulted in the mass confinement of the world’s population, the closure of country’s borders, the mass cancellation of flights, and the paralysis of business activities through temporary or permanent closure of non-essential services, leading to severe economic and social consequences worldwide (Alonso et al. 2020; Bapuji et al. 2020). Numerous studies have examined the impact of COVID-19 in different contexts, such as in business (Meyer et al. 2022; Genç 2021; Shen et al. 2020; Lin and Zhang 2020), in education (Genç and Köker 2021; Abumalloh et al. 2021; Chaturvedi et al. 2021), in mental health (Jones et al. 2021; Kola et al. 2021), or in tourism (Vaishar and Šťastná 2022; Senbeto and Hon 2020), with the tourism and leisure sector being one of the most affected (Robina-Ramírez et al. 2021; Duguleana and Duguleana 2020; Sigala 2020).

The tourism sector is very sensitive to risk scenarios caused by external factors, being strongly affected by economic crises, terrorism, natural disasters, or epidemic outbreaks (Kuo et al. 2008; Jiang et al. 2019). Crises in the tourism sector are common, for which many destinations have developed strategies. However, the COVID-19 pandemic is unlike any other, as it has led to a widespread collapse and reconfiguration of many segments of tourism supply and demand (Gössling et al. 2020; Kreiner and Ram 2020). Health restrictions and the fear of COVID-19 infection have negatively affected all countries, with the result that the tourism sector has never before faced such a major global crisis due to the sharp drop in global tourism demand (Yang et al. 2020; Nepal 2020; Chinazzi et al. 2020; Nguyen and Coca-Stefaniak 2020; Higgins-Desbiolles 2020a; Gössling et al. 2020).

According to United Nations World Trade Organisation (UNWTO 2021), the impact of the pandemic on international tourism has led to a 74% drop in demand, resulting in export revenue losses of USD 1.3 trillion, global Gross Domestic Product (GDP) losses of United States Dollars (USD) 2 trillion and losses of 120 million direct jobs. The most affected countries are those with the highest tourism activity (Sánchez-Cañizares et al. 2021), including Spain, given that tourism is essential to the countries economy, accounting for 1.8% of the countries GDP, and more than 52% of services exports (Organisation for Economic Cooperation and Development 2020). An example of this impact is the decline in international tourism in Spain, which lost 71.09% of tourists in 2020 compared to 2019 (Spanish National Statistics Institute 2021a). However, the sector has not behaved in the same way, with tourist destinations located in natural areas being less affected than those located in urban areas. This behaviour is due to a preference for outdoor activities, as an alternative to other more crowded destinations (Sánchez-Pérez et al. 2021) where the perception of risk is higher (Sánchez-Cañizares et al. 2021). For tourists, safety is paramount, and they are more reluctant to stay in hotels (Gursoy and Chi 2020; Richards and Morrill 2021). The changing trend, with a strong preference for nature destinations (Higgins-Desbiolles 2020a, b; Dachary et al. 2020; Sigala 2020; Carr 2020; Stankov et al. 2020) where outdoor activities are possible (Sánchez-Pérez et al. 2021), has an impact on regional economies and public health (Buckley et al. 2019). The growing territorial prominence of wilderness and travel restrictions due to the health crisis have provided an opportunity for outdoor tourism. Camping is considered an outdoor activity, being one of the best alternatives for nature-based tourism (Cohen 2020; Şengel et al. 2020). Camping involves at least one night away from home in accommodation such as a tent, vehicle, or caravan (Brochado and Pereira 2017; Lee et al. 2019). Camping tourism is determined by its inseparable relationship with the natural environment and the flexible, temporary, and mobile nature of its facilities (Blichfeldt and Mikkelsen 2015). Camping tourists associate camping tourism with freedom of access to natural spaces, tranquillity, self-sufficiency, and the absence of crowds (Kearns et al. 2019), which provide a pleasant form of accommodation (Hardy et al. 2012). For many years, this tourism has portrayed a lifestyle, presenting itself as consumer tourism that allows city dwellers to surround themselves with a natural environment in a short time thanks to nearby campsites (Timothy and Teye 2009). Camping has proven to be resilient to the effects of COVID-19, as pandemic adaptation measures have led to a rebound in the camping sector, becoming busier than usual (National Parks Service 2021). Tourism and nature-based recreational activities, such as camping, experienced significant growth during the COVID-19 pandemic compared to more traditional forms of accommodation (e.g., hotels) (Yu et al. 2021; Gossling et al. 2020; Kim and Lee 2020). For the duration of the pandemic, demand will continue for secluded tourism in wilderness destinations located in coastal areas, natural settings, or near rivers, as these destinations provide greater ease of control over socialisation (Craig and Karabas 2021; Craig 2020). Tourists have assumed that social distancing and outdoor leisure are now part of the new normal (Hong et al. 2020; Rice et al. 2020). This puts camping tourism in a good position compared to more traditional types of accommodation (Gössling et al. 2020; Ma et al. 2020), as social interaction is less common and, therefore, campers feel more confident and secure in this environment (Şengel et al. 2020). Some research studying the effects of COVID-19 on the camping sector shows that distance, understood as spatial proximity, has not influenced the choice of camping as a form of tourism (Craig et al. 2021a), as leisure travellers living where the incidence of the pandemic is highest are willing to travel further to camp (Craig et al. 2021b).

Although camping tourism represents a major part of the tourism industry, there is very little research into this form of tourism from the hospitality field (Brooker and Joppe 2014; Mikulić et al. 2017). This research has largely been developed in Western countries (Choi and Dawson 2002; Oh et al. 2007; O'Neill et al. 2010; Mikulić et al. 2017). These articles focus on the study of market segments, user experience, operations, and development (Brooker and Joppe 2014), and analyse the characteristics that determine the choice of campsite, as well as user satisfaction in terms of environment and experience, with the aim of implementing management strategies (see, for example, Oh et al. 2007; O'Neill et al. 2010; Gursoy and Chen 2012; Mikulić et al. 2017; Cheng-Fei 2020). Some of this research shows that camping tourism constitutes a complex system, where multiple factors (tangible and/or intangible) play an important role in the evaluation of the tourist experience (Meng et al. 2008). For example, Breiby (2014) shows that in nature tourism, the factors that measure the aesthetics of the area are the dimensions of harmony, variation, contrast, landscape, views, authenticity, art, and architecture, and these are the factors most appreciated by the traveller.

Although camping tourism is a global phenomenon, it is especially prevalent in the United States (Timothy and Teye 2009; Brooker and Joppe 2014; Young 2017), New Zealand (Kearns and Collins 2010; Collins et al. 2018), Australia (Caldicott and Scherrer 2013; Caldicott et al. 2014), and South Africa (Van Heerden 2010a, 2010b, 2020), although it is also an important tourism modality in different European countries, such as Denmark (Mikkelsen and Cohen 2015; Mikkelsen and Blichfeldt 2015), Germany (Doğantan and Emir 2019), Spain (García-Pozo et al. 2011; Saló et al. 2020), and the United Kingdom (Lashley 2015), although growing interest is also emerging in Asia (MacLeod 2017; Cheng-Fei 2020).

Research into the characterisation of camping tourism as well as the analysis of its impact remains scarce (Seabra et al. 2014). One of the reasons for this is that authors have focused on the identification of certain factors that establish tourist behaviour and have left aside other aspects that can influence said behaviour. Among the few studies on this topic is the work of Grzinic et al. (2010), where, in the region of Dalmatia, the diversification of the tourism product, and the quality of the camping available are identified as factors in the development of camping tourism. Milohnić et al. (2014) study the specific trends influencing the market demand for camping caravans, with the quality and innovation of accommodation being the factors ensuring this demand. The quality of infrastructure and close contact with nature also act as determinants of the tourist destination (Cheng-Fei 2020).

In terms of territorial analysis of tourism, tourist destinations located on the coast are the most popular, since sun-and-sand tourism has become a mass phenomenon where millions of people seek rest and recreation (García-Pozo et al. 2011; Ley-Vega et al. 2007; Jedrzejczak 2004). Campsites located in coastal areas are preferred (Saló et al. 2020; Kearns and Collins 2010). In fact, in certain countries, such as Spain, South Africa, New Zealand, Australia, and Turkey, the camping sector is mostly associated with sun-and-sand tourism (Doğantan et al. 2017; Rogerson and Rogerson 2019, 2020; Kearns and Collins 2010), having been the most frequently consumed for many years. However, in the twenty-first century, there seems to be a certain shift towards alternative, nature-based forms of tourism as a consequence of changing economic conditions, consumer behaviour, and technological development (Çelik et al. 2017; O'Neill et al. 2010). The value of natural areas has increased, with growing interest in recreational activities in nature (Sánchez-Pérez et al. 2021; Sánchez-Sánchez and Sánchez-Sánchez 2021a; Akama and Kieti 2003). Proof of this is that in a tourism market as important as the United States, camping tourism is principally related to nature tourism (Craig 2020; Timothy and Teye 2009).

In Spain, tourists show a general preference for sun-and-sand tourism (Saló et al. 2020), although nature tourism is now becoming increasingly prevalent (Sánchez-Sánchez and Sánchez-Sánchez 2021b). Hence, the research question arises as to whether this change in trend will also become a reality in camping tourism. Our main working hypothesis, therefore, is that the special context caused by the COVID-19 health crisis has led to a change of trend in the Spanish camping sector, in favour of camping destinations located in natural areas, to the detriment of those located along the coast, the geographical location, therefore, affects the impact of tourism on the camping sector. To carry out this analysis, a spatial analysis model is proposed in which two different geographical locations will be considered: on the one hand, campsites located in natural areas; on the other hand, campsites located in coastal areas. This will enable a comparison of results and possible changes in the trend of these tourist areas. This work contributes to the literature on tourism impact studies of the camping sector, and proposes an innovative model of geographical analysis, whose main contribution is the territorial units studied. The analysis focuses on tourist areas, defined by the Spanish National Statistics Institute (INE) as “an area formed by a set of municipalities in which tourism is specifically located”. These destinations are smaller geographical spaces than those examined so far in the literature, which is often aligned with territorial approaches that consider a national or regional perspective. Despite the importance and interest that the analysis of tourism impact in these areas may present, spatial research remains scarce (De Carlos Villamarín et al. 2016), although some works can be found that analyse tourism at the geographical level, for example, in terms of the country (Lozano and Gutiérrez 2011), or the region (Barros et al. 2011a, 2011b; Huang et al. 2012; Brida et al. 2012; Benito-López et al. 2014; Sellers-Rubio and Casado-Diaz 2018). Therefore, the objectives of this research are: on the one hand, characterise the Spanish camping sector by determining the factors that describe it, and analyse their impact as an economic alternative in the two geographical spaces under study. On the other hand, establish territorial groupings of tourist destinations according to their determining factors, and analyse their evolution in the face of the COVID-19 health crisis. To achieve these objectives, economic, tourism demand, tourism supply, and tourism performance variables will be used, to which Multivariate Analysis techniques will be applied. This methodology will make it possible to establish associations and examine latent structures in the data, which will allow a diagnosis to be made of the reality of the Spanish camping sector on the outbreak of COVID-19.

Understanding tourism behaviour during and after major crises such as COVID-19 is a fundamental aspect of planning and recovery of tourism destinations. At present, the behaviour of tourists and their perceptions of tourist destinations are determined by the health situation of the pandemic. Therefore, characterising the impact of camping tourism will allow tourism institutions and managers to carry out effective management and planning, favouring decision-making to establish possible measures for promotion, distribution of investments, actions on natural resources, creation, and/or maintenance of infrastructures (Leco et al. 2015; Gómez-Limón and García 2014; Deery et al. 2012; McIntyre 1993).

This paper is structured as follows. “The camping sector in Spain” studies the situation of the Spanish camping sector. “Methodology and Sources” presents the methodology and data sources used in the study. “Factor Analysis” presents the results of the analysis, showing the characterisation of camping tourism and the territorial grouping of tourist destinations in Spain. Cluster Analysis contains the discussion of the study. Finally, in “Results”, the conclusions of the study are presented.

## The camping sector in Spain

The descriptive analysis of the sector based on the data available from INE enables camping tourism activity in Spain to be analysed. According to the data from 2017 to 2021 (Table 1), camping tourism activity experienced an increase of 5.6% in the period from 2017 to 2019; however, the total number of users suffered a very significant mean decrease of 55.3% between 2019 and 2021, which shows the strong impact of the COVID-19 pandemic on tourism demand. The other types of tourist accommodation have also been greatly affected by the health crisis, especially rural tourism and hotels, with losses in the number of travellers between 2019 and 2021 of 70.2 and 68.9%, respectively. Note that camping in 2020 is the type of tourist accommodation least affected by the health crisis.

**Table 1:** Travellers using tourist accommodation in Spain per year (total and annual percentage change). Prepared by the authors with data from the INE

	2017	2018		2019		2020		2021	
	Total	Total	Variation (%)	Total	Variation (%)	Total	Variation (%)	Total	Variation (%)
Hotels	103,804,067	105,311,465	1.5	108,716,047	3.2	34,589,071	–68.2	33,864,614	–2.1
Camping	7,869,189	7,867,359	0.0	8,304,242	5.6	4,489,473	–45.9	3,710,701	–17.3
Rural tourism	4,049,974	4,260,669	5.2	4,421,397	3.8	2,082,908	–52.9	1,319,240	–36.7
Hostels	1,023,932	1,075,339	5.0	1,087,343	1.1	234,111	–78.5	472,301	101.7
Total	116,747,162	118,514,832	1.5	122,529,029	3.4	41,395,563	–66.2	39,366,856	–4.9

The availability of data for the year 2021 means that information for this period is only available up to the month of July

As for the categories of camping accommodation, they are all negatively affected in the evolution of the number of users during the pandemic years (see Table 2), with highly significant decreases in the number of visitors.

**Table 2:** Tourists using camping accommodation in Spain per category and year in Spain (total and percentage of annual variation) Prepared by the authors with data from the INE

	2017	2018		2019		2020		2021	
	Total	Total	Variation (%)	Total	Variation (%)	Total	Variation (%)	Total	Variation (%)
Luxury and 1st class	3,462,974	3,466,419	0.1	3,700,518	6.8	1,861,894	–49.7	1,609,152	–13.6
2nd category	3,692,090	3,678,464	–0.4	3,834,297	4.2	2,170,808	–43.4	1,745,920	–19.6
3rd category	714,127	722,476	1.2	769,428	6.5	456,770	–40.6	355,629	–22.1
Total travellers	7,869,191	7,867,359	0.0	8,304,243	5.6	4,489,472	–45.9	3,710,701	–17.3

The availability of data for the year 2021 means that information for this period is only available up to the month of July

The number of establishments in the Spanish camping sector is stable, with an average of 715 campsites operating in Spain in the last 5 years, offering an average of 464,789 bedplaces, distributed across 143,295 plots, for which an average of 6,464 employees is needed (see Table 3). In terms of the size of the establishments, an average of 648 bedplaces per campsite is recorded, divided into an average of 199 plots per campsite and employing an average of 9 workers in each establishment (INE  2021). The average stay is 5.6 nights per visitor, with an average total number of 33,265,404 overnight stays (Table 3). Note that the years affected by the COVID-19 pandemic have notably affected the camping sector (especially in 2020), with a significant decrease in the number of establishments, plots, bedplaces, overnight stays, and number of employees.

**Table 3:** Supply of accommodation, overnight stays, and campsite employees per year Prepared by the authors with data from the INE

	2017	2018	2019	2020	2021	Average
Establishments	765	768	774	527	743	715
Plots	153,383	154,644	154,448	105,360	148,641	143,295
Bedplaces	494,366	501,734	502,002	340,483	485,363	464,789
Average stay	5.5	5.5	5.4	5.7	5.8	5.6
Overnight stays	38,711,803	39,158,716	40,720,536	21,565,347	26,170,616	33,265,404
Employees	6593	6937	7233	4601	6958	6464

The availability of data for the year 2021 means that information for this period is only available up to the month of July

**Fig. 1:**
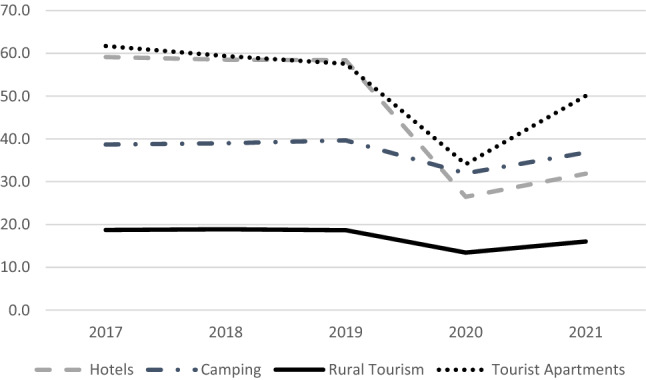
Average occupancy rate in tourist accommodation per year (%).*The availability of data for the year 2021 means that information for this period is only available up to the month of July. Prepared by the authors with data from the INE

The activity of many types of accommodation in the camping sector is strongly influenced by the weather and the holiday season, especially those located in coastal areas (García-Pozo et al., 2011). This means that the average occupancy rate per pitch is low, not exceeding 40% in the last 5 years, which contrasts with hotel occupancy rates, which are considerably higher than those of campsites (Fig. 1). Occupancy in any type of tourist accommodation has been notably affected by the COVID-19 health crisis, although camping and rural tourism have recorded considerably lower falls in occupancy than hotels and tourist flats (Fig. 1).

**Fig. 2:**
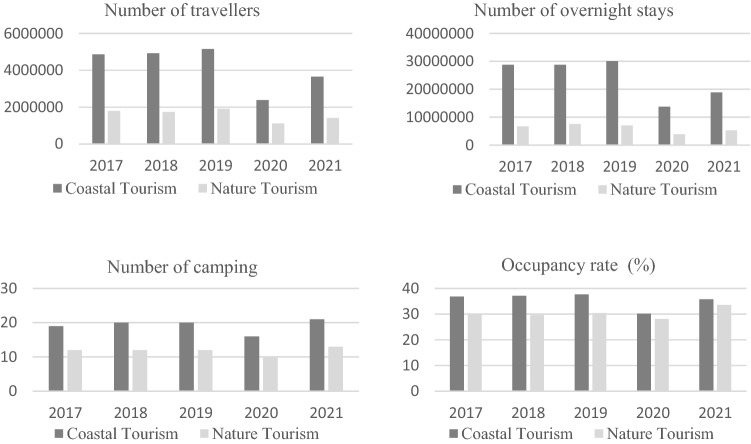
Number of travellers, overnight stays, camping sites, and occupancy rate per type of tourism (coastal and nature).*The availability of data for the year 2021 means that information for this period is only available up to the month of July. Prepared by the authors with data from the INE

A comparison between coastal and nature tourism by means of different indicators of the sector shows that, in terms of the number of travellers, overnight stays, and camping facilities, coastal tourism is the sector's favourite. However, in terms of occupancy rates, the figures for the two types of tourism are fairly similar and stable, with a slight upturn in the occupancy rates of nature tourism compared to coastal tourism in the years affected by COVID-19 (see Fig. 2).

## Methodology and Sources

The data considered for the analysis originates from official statistics, specifically from the Campsite Occupancy Survey, published by the INE. To study the impact of the pandemic on the camping sector, the years 2019 and 2020 are considered as the study period.

The INE defines a tourist area as “an area made up of a group of municipalities in which tourism is specifically located”. Therefore, tourist areas located both in coastal areas and in natural spaces are considered. The choice of study variables is conditioned by the availability of information on tourist areas in the databases. The study considers a total of 39 Spanish tourist areas in the camping sector, 17 of which are located in coastal areas, and 22 in natural areas.

For the selection of variables, it is considered that tourism is shown in terms of tourist supply and demand, infrastructures, resources, and the economic impact it generates. Thus, ten variables have been selected, which can be grouped into four thematic blocks (the unit of measurement of each variable is indicated in brackets)

1. Economy: staff employed (number of people).
2. Tourist demand: travellers (number of people), occupied plots (number of plots with tourists staying), overnight stays (number of overnight stays), and length of stay (number of days).
3. Tourist supply: plots (number of plots), bedplaces (number of bedplaces offered), and establishments (number of campsites).
4. Tourist Performance: occupancy rate per plot (%) and occupancy rate of plots at weekends (%).
5. The disparity of the units of measurement of the selected variables makes it necessary to standardise the data, which will enable the results to be presented in a relative way, thereby making them comparable.

Multivariate analysis will be used for data analysis in the form of the statistical techniques of Factor Analysis and Cluster Analysis. This methodology allows a better understanding of the phenomenon under study, providing information that univariate and bivariate that univariate and bivariate methods are unable to achieve. These methods allow to establish associations and operational laws, to examine latent structures and to deal with different ways of constituting data in known distributions. In addition, Multivariate Analysis is a useful technique for handling large databases, allowing for easy interpretation of large volumes of information, as in our case. This methodology is widely used in the literature to describe and represent areas in different contexts. For example, in the tourism sector, the work of Sánchez-Sánchez and Sánchez-Sánchez (2021a) utilises these techniques to identify and characterise determinants of rural tourism in protected natural areas in Spain, and analyzes their economic impact on the development of rural areas. Sánchez and Sánchez (2018) apply this methodology to study the effect of rural tourism on employment in Spain. Fernández-Morales and Mayorga-Toledano (2018) use multivariate analysis to analyse cruise tourism, and establish territorial groups according to the incidence of this type of tourism. De Carlos Villamarín et al. (2016) apply this methodology to study the performance of various tourist destinations along the Spanish coast. Other studies in which these techniques are applied in contexts other than tourism include those of Serra et al. (2014), Sánchez et al. (2018), Cruces Pastor et al. (2009), Pena López and Sánchez Santos (2008), and Herrero Prieto et al. (2007). Other international research that uses multivariate methodology to identify and describe geographical units are, for example: in Tanzania (Jani 2018), in Ecuador (Santamaría-Freire et al. 2017), in Nigeria (Agbabiaka et al. 2017), in Portugal (Vareiro et al. 2013), in Romania (Dona and Popa 2013), and in Italy (Brida et al. 2010).

### Factor Analysis

Factor analysis seeks to determine factors (also called dimensions) with which to explain the correlations between variables. To this end, the original information of the variables is summarised with compositions of these dimensions, which gives rise to the factors sought. This technique makes it possible to obtain a smaller number of latent variables (the so-called factors) that collect the information of the subjects studied in the simplest possible way.

Factor analysis uses a linear model, with which it associates variables with factors, formulating the original observed variables as a linear combination of unobserved factors, according to the following expression:

1 $$Y_{ij} = \mathop \sum \limits_{k = 1}^{m} \beta_{ik} F_{kj} + E_{ij} ,$$

where *Y*_*ij*_ is the value of the variable *Y*_*i*_ in tourist area *j*; *F*_*kj*_ is the value of factor *k* in tourist area *j*; *E*_*ij*_ captures the part of *Y*_*i*_ in tourist area *j* that is not explained by the factors in the model; and where *β*_*ik*_ is the standardised regression coefficient of the variable *Y*_*i*_ on the factor *F*_*k*_, which measures the weight of each factor through the proximity between the factor *F*_*k*_ and the variable *Y*_*i*_. Therefore, the higher the coefficient of a factor becomes, the greater the relationship grows between said factor and the corresponding variable.

The analysis to be applied will use an exploratory and non-inferential approach, and employs the Principal Components method (Morrison 1987) for the extraction of factors and sets the selection of those that obtain an eigenvalue greater than unity as a criterion (Kaiser 1960).

### Cluster Analysis

Once the factors describing the different tourist areas have been extracted, they are utilised to carry out the Cluster Analysis, which identifies territorial groups of tourist areas with similar characteristics in the factors. Cluster Analysis classifies different individuals into groups (clusters), based on the particularities they show, such that the individuals belonging to a cluster are as similar as possible to each other and those in different clusters are as different as possible to those in the first cluster (Hair et al. 2000). To determine the clusters, the idea of similarity between individuals is used, which is mathematically measured by “distance” One possible way of measuring distance is through the Euclidean distance squared. There are other alternatives for measuring distance, but as Hair et al. (2000) show, the selected measure does not significantly affect the result obtained.

There are two possible methods for cluster construction: hierarchical and non-hierarchical. Hierarchical methods assume all possible sets, while non-hierarchical methods set an initial number of clusters. In our analysis, first, the appropriate number of clusters will be determined, and then, the non-hierarchical k-means method will be used.

## Results

The organisation of the results is shown in the same order as the objectives of the study: (1) identification and definition of the factors that describe the tourism sector of the campsite; (2) determination of territorial groups of tourist destinations according to their characterising factors.

### Characterisation of Camping Tourism

First, the factors that characterise camping tourism in the main Spanish tourist destinations in 2019 and 2020 are determined. The time period considered for the study will allow a comparison of the impact of the COVID-19 pandemic on the camping tourism sector.

We begin by studying the feasibility of the application of factor analysis, through the study of Bartlett's test of sphericity, which will enable the verification of whether the factor model is applicable, by checking whether the correlation matrix of the variables is an identity matrix. For the years 2019 and 2020, the analysis provides a Bartlett’s statistic of 900.199 and 748.403, respectively, and a small significance level (0.0000 in both cases), which indicates that the application of Factor Analysis is suitable. This result is confirmed by the Kaiser–Meyer–Olkin coefficient: 0.749 for 2019, and 0.700 for 2020.

Regarding the selection of eigenvalues, those that are greater than unity will be chosen. This criterion allows two factors to be selected, with an explanatory capacity in 2019 of 89.501% of the total variability and 86.106% in 2020 (see Table 4). The high percentages explained confirm the goodness of fit of the selected models, since, in studies related to Social Sciences, the lower limit of acceptance is 60% (Hair et al. 2000).

**Table 4:** Determinants of tourist destinations and explained variance Authors’ own

Factors	Year 2019			Year 2020		
	Eigenvalues	% Of variance	% Accumulated	Eigenvalues	% Of variance	% Accumulated
Factor 1. Tourism supply–demand balance and labour dynamism	6.925	69.253	69.253	6.484	64.836	64.836
Factor 2. Effectiveness of camping tourism	2.025	20.248	89.501	2.127	21.271	86.106

Tables 5, 6 show, in decreasing order, the values of the coefficients of the rotated factor matrix for Factors 1 and 2, in the years 2019 and 2020, respectively. These scores record the weight of each variable in the factor, whereby the higher the factor score, the greater the established relationship.

**Table 5:** Rotated factorial matrix of Factor 1 according to year

Year 2019		Year 2020	
Variables	Factor Score	Variables	Factor Score
Plots	0.984	Plots	0.985
Bedplaces	0.982	Bedplaces	0.974
Travellers	0.974	Staff employed	0.969
Staff employed	0.958	Travellers	0.941
Overnight stays	0.94	Overnight stays	0.938
Occupied plots	0.928	Occupied plots	0.914
Establishments	0.817	Establishments	0.827
Stay	0.186	Stay	0.203
Occupancy rate of plots at weekends	0.158	Occupancy rate per plot	0.07
Occupancy rate per plot	0.134	Occupancy rate of plots at weekends	0.016

**Table 6:** Rotated factorial matrix of Factor 2 according to year Authors’ own

Year 2019		Year 2020	
Variables	Factor Score	Variables	Factor Score
Occupancy rate per plot	0.952	Occupancy rate per plot	0.964
Occupancy rate of plots at weekends	0.949	Occupancy rate of plots at weekends	0.953
Stay	0.741	Stay	0.592
Occupied plots	0.35	Occupied plots	0.354
Overnight stays	0.261	Overnight stays	0.250
Staff employed	0.206	Establishments	0.178
Establishments	0.163	Staff employed	0.078
Plots	0.149	Plots	0.073
Bedplaces	0.138	Bedplaces	0.045
Travellers	0.048	Travellers	-0.007

In the years 2019 and 2020, Factor 1 explains 69.253 and 64.836% of the total variability (see Table 4), with which seven of the ten variables studied are strongly associated: Plots, bedplaces, travellers, employed staff, overnight stays, occupied plots, and establishments. These associations are determined by the positive correlation between the variables (Table 5), indicating that high (or low) values of Factor 1 are related to tourist destinations with high (or low) values of plots, bedplaces, travellers, staff employed, overnight stays, occupied plots, and establishments. Since not only the variables that determine Factor 1 are considered, where aspects related to the supply and demand of camping tourism resources stand out, but also their labour impact, this factor is labelled as Tourism supply–demand balance and labour dynamism. In 2019, the tourist areas with the highest scores in Factor 1, which measures the relationship between camping tourism supply and demand and labour dynamism, included, on the one hand, the tourist areas located on the Costa Brava with 4.38 points in 2019 and 3.70 points in 2020, and on the other hand, the Costa Dorada with 2.47 points in 2019 and 2.27 points in 2020.

Factor 2 explains 20.248% of the total variance in 2019 and 21.271% in 2020 (see Table 4), and is strongly and positively related to three of the variables analysed: occupancy rate per plot, occupancy rate of plots at weekends, and stay (see Table 6). These relationships indicate that high (or low) values of Factor 2 are associated with tourist destinations with high (or low) values of occupancy rate per plot, occupancy rate of plots at weekends, and days of stay. Therefore, Factor 2 is labelled as effectiveness of camping tourism. The areas with the highest scores in Factor 2 are Costa Blanca with 3.56 points and 3.30 points in 2019 and 2020, respectively, and the Serra Calderona Natural Park with 1.93 points in 2019 and 2.63 points in 2020.

The results obtained indicate that the relationship between supply and demand in the Spanish camping sector is significant, since it a positive impact on the economy of certain tourist destinations, especially in terms of the labour market. Another important aspect that emerges from the analysis involves the good results obtained with regard to the performance of the sector, as the relationship between occupancy and tourist production indicates. With regard to the impact of the health crisis on the factors identified, it does not seem that the pandemic significantly modifies the determinants that characterise the sector, as the results obtained during the period of analysis remain similar.

## Territorial Grouping of Tourist Destinations

The determination of the different groups (clusters) of tourist destinations will allow the identification of tourist areas with similar characteristics, in accordance with the previously determined factors. The non-hierarchical method will be used to create the clusters, using the different scores obtained in the tourist areas for the two previously identified factors. To establish the appropriate number of clusters, various tests have been carried out for different numbers of clusters, and for these, the deviations that occur in the residual variance have been evaluated. Based on these variations, it was decided to select six clusters as the optimal number. Table 7 shows the territorial configuration obtained in the different clusters for the years 2019 and 2020.

**Table 7:** Configuration of tourist destinations according to cluster and year Authors’ own

	Year 2019
Cluster	Tourist Destinations
1	24 Zones. Costa de Almería, Costa de la Luz de Cádiz, Costa Tropical, Costa de la Luz de Huelva, Costa Verde, Terres de l'Ebre, Norte de Extremadura, Rías Altas, Rías Baixas, Costa Guipuzkoa, Pirineo Aragonés, Pirineo Navarro, Parque Nacional Sierra Nevada, Parque Nacional Ordesa, Parque Nacional Aigüestortes, Parque Natural Doñana, Parque Natural Los Alcornocales, Parque Natural Sierra de Grazalema, Parque Natural Sierra de Hornachuelos, Parque Natural Sierra Nevada, Parque Natural Sierras de Cazorla, Segura y las Villas, Parque Natural Sierra de las Nieves, Parque Natural Sierra y Cañones de Guara, Parque Natural Alt Pirineu
2	8 Zones. Costa del Sol, Costa Valencia, Costa Cálida, Costa Bizkaia, Parque Nacional de Sierra de Guadarrama, Parque Natural Zona Volcánica de la Garrotxa, Parque Natural Cadí-Moixeró, Parque Natural Serra Calderona
3	4 Zones. Costa Barcelona, Costa de Castellón, Pirineus, Parque Natural de los Aiguamolls de I'Empordá
4	1 zone. Costa Brava
5	1 zone. Costa Dorada
6	1 Zone. Costa Blanca

Cluster	Year 2020
	Tourist Destinations
1	16 Zones. Costa de Almería, Costa del Sol, Costa Valencia, Rías Altas, Costa Cálida, Costa Guipuzkoa, Costa Bizkaia, Pirineo Navarro, Parque Nacional Ordesa, Parque Nacional Aigüestortes, Parque Natural Los Alcornocales, Parque Nacional de Sierra de Guadarrama, Parque Natural Sierra y Cañones de Guara, Parque Natural Zona Volcánica de la Garrotxa, Parque Natural Cadí-Moixeró, P. Natural Alt Pirineu
2	14 Zones. Costa de La Luz de Cádiz, Costa Tropical, Costa de La Luz de Huelva, Costa Verde, Terres de l'Ebre, Norte de Extremadura, Rías Baixas, Parque Nacional Sierra Nevada, Parque Natural Doñana, Parque Natural Sierra de Grazalema, Parque Natural Sierra de Hornachuelos, Parque Natural Sierra Nevada, Parque Natural Sierras de Cazorla, Segura y las Villas, Parque Natural Sierra de las Nieves
3	2 Zones. Costa Brava, Costa Dorada
4	1 Zone. Costa Blanca
5	5 Zones. Parque Natural de los Aiguamolls de I'Empordá, Pirineo Aragonés, Pirineus, Costa de Castellón, Costa Barcelona
6	1 Zone. Parque Natural Serra Calderona

Table 8 shows the final mean scores of the clusters in each of the factors for the years 2019 and 2020, which enables the importance of these clusters to be quantified in each of the extracted factors.

**Table 8:** Average scores in the clusters per year Authors’ own

Factors	Cluster (Year 2019)					
	1	2	3	4	5	6
Factor 1. Tourism supply–demand balance and labour dynamism	–0.27349	–0.61695	0.88822	4.38011	2.47331	1.09302
Factor 2. Effectiveness of camping tourism	–0.55665	1.05473	0.50461	–0.77063	0.10646	3.56736

Factors	Cluster (Year 2020)					
	1	2	3	4	5	6
Factor 1. Tourism supply–demand balance and labour dynamism	–0.46732	–0.30754	2.98768	1.87934	1.02797	–1.21183
Factor 2. Effectiveness of camping tourism	0.41574	–0.80322	–0.57313	3.30819	–0.04574	2.65995

In 2019, in Factor 1, which measures the relationship between supply and demand in the sector together with labour dynamism, Clusters 4 and 5 stand out (see Table 8), made up of the Costa Brava and Costa Dorada (Table 7), respectively, both destinations located on the Catalan coast. This highlights the weight of camping tourism in Catalonia, as well as the traveller's preference for sun-and-sand tourism. However, in the year 2020, in Factor 1, there are several significant changes in terms of the clusters highlighted in this factor, which incorporates tourist destinations located both in coastal areas and in natural spaces. Specifically, in Factor 1, Cluster 3, made up of the Costa Brava and Costa Dorada, Cluster 4, made up of the Costa Blanca, and Cluster 5, made up of the Aiguamolls de l'Empordá Natural Park, Aragonese Pyrenees, Pyrenees, the Castellón Coast, and the Barcelona Coast (Table 7), carry significant weight (Table 7). This result shows the diversification of the impact on the supply and demand of tourist areas, as well as the greater weight of nature tourism, which is evidence of a certain change of trend in the preferences of the type of tourism in Spain in the year 2020, with an evolution in the preferences of tourists towards natural areas.

In 2019, in Factor 2, which measures tourism efficiency, Cluster 6 (comprising the Costa Blanca) and Cluster 2 (comprising the Natural Parks of Serra Calderona, Cadí-Moixeró, the Garrotxa Volcanic Zone, the Sierra de Guadarrama National Park, Costa Bizkaia, Costa Cálida, Costa Del Sol, and Costa Valencia) stand out. The evolution of this factor for the year 2020 shows Cluster 4 (Costa Blanca) and Cluster 6 (Serra Calderona Natural Park) as outstanding, which reveals a loss of tourist efficiency in both coastal destinations and natural areas, which in turn highlights a non-optimal use of available resources in the camping sector. This loss may be due to the significant drop in tourism demand during the pandemic, to the strong health restrictions imposed to control the virus, to the availability of resources, and/or to infrastructures.

The rest of the clusters, for any of the years analysed, have low or below-average scores in the factors extracted, which indicates that these clusters do not stand out in any factor (Table 8). These clusters are numerous and heterogeneous, comprising both coastal destinations and natural areas (Table 7). This shows the uneven impact of tourism on the Spanish camping sector, since few coastal areas and those located in natural areas stand out in either of the factors.

The results obtained show that Spain is a country where the camping tourist especially prefers coastal areas, with the Catalan and Valencian coasts as the most popular locations. However, the COVID-19 health crisis shows a certain change of trend in the preferences of tourists, who are increasingly opting for nature tourism. However, it might be expected, perhaps, that this change would be more noticeable than the results show. It may be that this result is influenced by the type of tourism studied, given that camping is a type of tourism that offers freedom of access to natural areas, absence of crowds, tranquillity, self-sufficiency, and social distancing, both in coastal destinations and in natural areas.

## Discussion

The Spanish camping tourism map is asymmetrical, and presents a strong concentration of supply and demand for camping tourism in coastal areas, where the hegemony of this type of tourism is established, especially along the Catalan coast (Baños and Rico 2016; López Palomeque 2009), where the impact of COVID-19 has barely exerted any negative effect on this type of tourism, with a fairly stable supply and demand, which is evidence of the loyalty of camping tourists (Triantafillidou and Siomkos 2013; Kearns and Collins 2010). This demonstrates the importance of the spatial approach for the characterisation carried out, as the fact of studying smaller territorial units than those usually used in the literature (e.g., countries, regions) makes it possible to identify areas of tourist influence of campsites, which will allow managers and authorities to improve the understanding of tourism systems, favouring decision-making and the development of strategies for specific tourist destinations. This shows the importance of the characteristics of the tourist destination, given that the variables used to measure both tourism supply and demand are mainly determined by the geographical area (tourist zone), a result similar to that obtained by Marco-Lajara et al. (2016). The resources associated with the territory make some tourist areas more attractive than others. In this sense, it seems that the COVID-19 pandemic is a determining factor in the fact that natural areas are an important resource in characterising the importance of campsites located in these environments as opposed to those located in coastal areas.

The impact of the COVID-19 health crisis on the tourism sector has been global, despite the fact that certain types of tourism have been more affected than others, such as those involving large crowds of tourists (such as tourism in large cities and recreational sun-and-sand tourism). In Spain, there has been a certain change in the preferences of camping tourists, showing that natural areas have been able to generate their own tourism dynamics (Vera et al. 2011), and are favoured over coastal destinations. Despite presenting such a change, its effect is not as significant as might be initially expected. This may be due to camper’s resistance to change, since the perception of change may be considered as a threat to their experience (Walsh and Lipinski 2008). Another possible cause is the very restrictions caused by the pandemic (perimeter closures, mobility restrictions, etc.), which have favoured the development of local forms of travel, thereby forcing a closer type of tourism, and focusing on national and regional markets (Bieger and Laesser 2020; Dupeyras et al. 2020; Gössling et al. 2020; Rogerson and Baum 2020).

As in other studies, the resilience of the camping sector to the COVID-19 pandemic has been shown (Yu et al. 2021; Gossling et al. 2020; Kim and Lee 2020), as it is able to show very positive results, especially in economic aspects (employment and sector performance). It is to be expected that, for the duration of the pandemic, the demand for outdoor “isolation” tourism will continue, as it is easier to control socialisation in these destinations. All this means that camping tourism has good prospects for the future.

## Conclusions

This paper studies camping tourism, which is associated with outdoor recreational tourism. The study is motivated by the importance and weight of tourism in the Spanish economy, and aims to study the impact of the COVID-19 pandemic on the camping tourism modality, since, due to its special characteristics, this tourism modality may have more adequate resources than other tourism modalities to face the health crisis. This work contributes to the literature on tourism impact, and proposes an innovative model of geographical analysis, whose main contribution is the territorial units studied. Its analysis focuses on tourist areas located on the one hand on the coast and on the other in natural areas, which allows the comparison of results in two areas in which two types of tourism with different characteristics are developed. The tourist destinations proposed as a unit of analysis are smaller geographical spaces than those examined so far in the literature.

With regard to the hypotheses and objectives set out, the analysis of camping tourism in Spain allows the following conclusions to be drawn:

(a) Two determinants of camping tourism have been identified. The tourism supply–demand balance and labour dynamism factor is characterised by the variables number of plots, bedplaces, travellers, staff employed, overnight stays, occupied plots and establishments, showing the significant and balanced relationship between supply and demand in the sector, as well as the significant weight of employment it generates. Before the pandemic, campsites located in coastal areas were the most important in this factor, with a majority demand from the camping tourism market, with the coasts of Catalonia and Valencia being the tourist's favourites. However, the health crisis has led to nature tourism gaining greater notoriety in this factor, showing an evolution in the preferences of camping tourists towards campsites located in natural areas, which is evidence of a change in tourist habits. This result may be influenced by the perception of risk offered by the tourist destination itself, which means that tourists avoid overcrowded destinations and look for open natural spaces, generally with few crowds of people, meaning that destinations located in natural areas are less affected by the crisis than those located in coastal areas. The second factor characterises the effectiveness of camping tourism, identifying as important aspects of the factor, the occupancy rate per plot, the occupancy rate of plots at weekends and stay, i.e., the performance of the sector, which is measured through the results of occupancy and tourist production. The pandemic causes an evolution of the most outstanding areas in the factor, with the natural areas benefiting the most from the efficiency of the sector in the COVID-19 period. However, it should be pointed out that it is evident that the health crisis has caused the camping sector, regardless of its geographical location, to show a notable decrease in tourism performance, reflecting the fact that the availability of resources in the sector does not guarantee their optimal use. The fact that an area has more tourism resources does not guarantee efficiency, and it is the good use or exploitation of these resources that comes into play. These results show that, in terms of tourism performance, there is still work to be done in the Spanish camping sector.

(b) The territorial grouping shows a certain shift in tourist preferences towards campsites located in natural areas. This confirms the research hypothesis that established that the COVID-19 pandemic would produce a change in preferences in the Spanish camping tourism modality, with campsites located in natural areas being favoured, to the detriment of coastal campsites. Therefore, geographical location is a determining factor in the impact of tourism on the type of campsite. The proposed spatial analysis model is useful to identify territorial areas of tourist influence of Spanish camping. The geographical effect of camping tourism in Spain is quite heterogeneous in the different tourist areas, given the diversity of groups of tourist areas determined by the factors.

The above analysis shows that, despite the fact that uncertainty continues to dominate the tourism sector, camping tourism in Spain represents an important market segment, being a type of tourism that has withstood the health crisis well, becoming consolidated during the COVID-19 pandemic. The empirical results show the need for managers and institutions of tourist destinations to understand the importance of the camping sector and its impact on tourist destinations. Such understanding and learning can help in making decisions to deal with future crises. In terms of practical implications arising from the study, these can relate both to the camping enterprises themselves and to the institutions. Regarding the former, when companies have to decide on their location, they should be aware that some locations may be more attractive than others due to the resources associated with the territory, given that in some areas, tourism has a greater impact on campsites located in coastal areas and in others on those located in natural spaces. As far as institutions are concerned, local governments of tourist destinations should adopt different policies to support the tourism sector, so that the different companies related to the camping sector are profitable, competitive, and have incentives to set up in their territory.

The main limitation of the study is the lack of official data on the opinion of camping tourists regarding the tourist areas analysed. This information would undoubtedly have enriched the study, as it would have made it possible to identify the factors that determine tourist behaviour, as well as the aspects that can influence their behaviour.

For future research, the model proposed here can be validated in other countries where the demand for camping tourism is similar to that of Spain, given that they are direct competitors of the country and its analysis can help in the planning of tourism strategies in relation to these countries. The proposed model can also be extended by considering other geographical units of analysis, which could be smaller territorial units than those considered here, such as, for example, cities, municipalities, tourist points, etc. However, it should be noted that this may present an important limitation, which is the lack of data for such small territorial units.
